# Pralatrexate is a potent pan-serotype human adenovirus inhibitor through suppression of dihydrofolate reductase

**DOI:** 10.1128/aac.01960-25

**Published:** 2026-05-28

**Authors:** Xiaowen Liang, Yong Feng, Chang Xu, Yuelin Wang, Wei Yang, Jiahua Kuang, Yanling Luo, Weihua Wu, Fangfang Chang, Lifeng Fu, Yingxia Liu, Chunmei Jiang, Liuqing Yang, Fuxiang Wang, Yang Yang

**Affiliations:** 1 School of Pharmacy, Shenzhen University Medical School, Shenzhen Unversity 481870, Shenzhen, Guangdong, China; 2 Shenzhen Key Laboratory of Pathogen and Immunity, Shenzhen Third People's Hospital, Second Affiliated Hospital, School of Medicine, Southern University of Science and Technology 535206, Shenzhen, China; 3 National Clinical Research Center for Infectious Diseases 638560 , Shenzhen, China; 4 Laboratory of Pathogen Microbiology and Immunology, Institute of Microbiology, Chinese Academy of Sciences 74519, Beijing, China; 5 University of Chinese Academy of Sciences 74519, Beijing, China; 6 Department of Infectious Disease, The People's Hospital of Longhua 380381 , Shenzhen, China; Chinese Academy of Medical Sciences & Peking Union Medical College, Beijing, China

**Keywords:** human adenovirus, pralatrexate, broad-spectrum, 3D lung organoid, DHFR

## Abstract

Human adenovirus (HAdV) is one of the most infectious pathogens that can cause diseases affecting multiple organ systems, posing a significant threat to public health. Currently, there are no specific antiviral therapies available for HAdV infection. In this study, three folate antagonists aminopterin (AMT), pralatrexate (PDX), and methotrexate (MTX) with potent antiviral activity against HAdV infection have been identified through a drug repurposing screening strategy. The three drugs showed broad and potent antiviral efficiency against multiple clinically prevalent HAdV serotypes, including HAdV-B3, HAdV-B7, HAdV-B55, HAdV-C2, and HAdV-C5 with half-maximal effective concentration (EC_50_) values ranging from 0.31 to 79.74 nM, and PDX further showed similar antiviral efficiency against HAdV-B55 in a 3D lung organoid model (EC_50_ = 0.51 nM). Mechanistic studies revealed that PDX achieved its antiviral activity through suppression of dihydrofolate reductase (DHFR). These findings support the potential of folate antagonists as repurposed therapeutic agents against HAdV infection and provide a rationale for the development of host-directed antiviral strategies.

## INTRODUCTION

Human adenovirus (HAdV) is a non-enveloped, double-stranded DNA virus (1). To date, more than 110 genotypes of HAdV have been identified and categorized into 7 species (A–G) (1). HAdV infections are highly prevalent in humans and can affect multiple organ systems, including the respiratory, digestive, urinary, and nervous systems (2). In immunocompetent individuals, HAdV infections are often mild and self-limiting. However, among immunocompromised populations, HAdV infections are highly prone to progression to severe disease with high mortality rates (3, 4). Notably, Human adenovirus type 55 (HAdV-B55) has attracted significant attention due to its notably high pathogenicity and clinical severity (5, 6). HAdV-B55 can cause severe and fatal pneumonia in children and immunocompetent adults, characterized by clinical features such as high fever, cough, respiratory failure, and bilateral lung consolidation (6–8).

Currently, no targeted antiviral therapeutics are available for HAdV infection. Cidofovir (CDV) exhibits anti-adenoviral activity through inhibition of viral DNA polymerase (9). However, its clinical application is constrained by dose-limiting nephrotoxicity and poor oral bioavailability (2). Brincidofovir (BCV) is a lipid-conjugated prodrug of CDV (10). It exhibits improved cellular uptake and metabolic stability, substantially reducing renal toxicity and enabling oral administration. Nonetheless, gastrointestinal adverse effects remain a significant dose-limiting concern (2, 10). Consequently, there is an urgent need to develop effective and safe treatment strategies against HAdV.

Host-directed therapy offers a new direction for drug development against HAdV infection. By targeting host cellular factors or pathways essential for viral replication, this strategy can not only overcome the common problem of drug resistance associated with direct-acting antivirals (DAAs) but also hold potential for developing broad-spectrum inhibitors due to its action on genetically stable host targets (11, 12). Given the extensive serotypic diversity and significant inter-type variations of HAdV, therapies targeting single viral proteins are often limited by narrow coverage and a tendency to induce serotype-specific resistance (1). Therefore, screening compounds that interfere with host pathways commonly relied upon by HAdV is an effective strategy for discovering broad-spectrum anti-HAdV drug candidates.

Due to the strict species specificity of HAdV, research has long been constrained by the lack of ideal models that accurately mimic human infection processes (13). Traditional two-dimensional (2D) cell lines fail to recapitulate the complex cellular diversity and structural functions of the human respiratory tract. Although transgenic mouse models permit infection, they do not support efficient viral replication and cannot accurately mimic the human immune response (14). Tree shrews can support the effective replication of HAdV species B and develop severe interstitial pneumonia after infection (15). However, as laboratory animals, they have disadvantages such as difficult acquisition, cumbersome procedures, and high costs (13). In recent years, the development of human organoids has exerted a significant influence on clinics and research (16). The National Institutes of Health (NIH) announced the establishment of a National Standardized Organoid Modeling Center (SOM) in September 2025 (17). This initiative will promote a shift in clinical research from reliance on animal models toward organoid models based on human biology. Organoids are generated from human pluripotent stem cells or primary cells cultured in three-dimensional (3D) conditions to form self-organizing microtissues that better preserve the cellular heterogeneity and functional characteristics of the originating tissue (18). Organoids can more accurately mimic the structure and function of human organs, thereby effectively overcoming the species differences inherent in traditional animal models and significantly improving the predictability of drug responses (16, 18).

In this study, we developed and validated a cytopathic effect (CPE)-based drug repurposing screening workflow for the identification of potential antiviral drugs against HAdV. Based on a library of 3,924 host-targeting compounds, we identified three folate antagonists that effectively inhibit different serotypes of HAdVs. These compounds, specifically aminopterin (AMT), pralatrexate (PDX), and methotrexate (MTX), all function as dihydrofolate reductase (DHFR) inhibitors. Among them, PDX further demonstrated significant antiviral activity in the 3D lung organoid model, underscoring its potential as a novel therapeutic option against HAdV infection.

## MATERIALS AND METHODS

### Cell and virus culture

HEp-2 cell, A549 cell, and 293T cell were obtained from the American Type Culture Collection (ATCC) and maintained in Dulbecco’s modified Eagle’s medium (DMEM) (Gibco, Cat. # C11995500BT) supplemented with 10% fetal bovine serum (FBS) and 1% penicillin-streptomycin (Gibco, Cat. # 15140122). All cells were cultured at 37 °C in a 5% CO₂ atmosphere. The viral strains used in this study, including HAdV-B55, HAdV-B3, HAdV-B7, HAdV-C2, and HAdV-C5, were isolated from clinical samples and confirmed by sequencing. Viruses were propagated in HEp-2 cells cultured in DMEM containing 2% FBS and 1% penicillin-streptomycin. When HEp-2 cells reached 100% confluency, they were infected with HAdV at a multiplicity of infection (MOI) of 0.5. At 48 h post-infection (hpi), the culture medium was collected and centrifuged at 4,000 rpm for 20 min. The supernatant was then aliquoted and stored at −80°C.

### Compound library and screen

A library of 3,924 compounds maintained in our laboratory was utilized for the screening. All compounds were provided as 10 mM solutions in dimethyl sulfoxide (DMSO) and stored at −20 °C until use. HEp-2 cells were seeded in 96-well plates at a density of 2.5 × 10⁴ cells per well on the day prior to infection. The following day, HAdV-B55 virus was mixed with the test compounds, and 100 μL of the mixture was added to each well, resulting in a final compound concentration of 10 μM and a multiplicity of infection MOI of 0.5. Each plate included a positive control group (treated with CDV), a negative control group (virus-infected without compound), and a blank control group (uninfected and untreated cells). When extensive CPE was observed at 96 hpi, the supernatant was aspirated, and the cells were gently washed with PBS. Then, 100 μL of 1.5% neutral red medium was added to each well, and the plates were incubated at 37 °C with 5% CO₂ for 4 h. After incubation, the neutral red medium was removed, and the cells were washed with PBS. Subsequently, 100 μL of destaining solution (50% ethanol, 49% deionized water, 1% glacial acetic acid) was added to each well. The absorbance at 540 nm (OD_540_) was measured using a Synergy H1 multifunction microplate reader (19). The percentage inhibition was calculated as follows: Inhibition (%) = [(OD_540_ of compound-treated group − OD_540_ of negative control)/(OD_540_ of blank control − OD_540_ of negative control)] × 100%. In subsequent studies, all compounds were purchased from MedChemExpress (MCE). The half-maximal effective concentration (EC_50_) and the half-maximal cytotoxic concentration (CC_50_) were calculated from the dose-response curve using GraphPad Prism 10.0. The selection index (SI) was calculated as CC_50_/ EC_50_.

### Quantitative real-time PCR

Viral genome DNA was extracted using a commercial nucleic acid extraction kit (Huayin Biotech, Cat. # DW-8516). Quantitative real-time PCR was performed using the TransScript Probe One-Step qRT-PCR SuperMix kit (TransGen Biotech, Cat. # AQ221-02) on a Life Technologies QuantStudio 7 Flex system. Each 20 μL reaction contained 10 μL of 2 × PerfectStart Probe One-Step qPCR SuperMix, 0.4 μL each of forward primer (10 μM), reverse primer (10 μM), and probe (10 μM), 0.4 μL TransScript Probe One-Step RT/RI Enzyme Mix, 5.4 μL RNase-free water, and 3 μL RNA template.

The primers and probe used for detection were as follows: Forward: 5′-GCCACGGTGGGGTTTCTAAACTT-3′; Reverse: 5′-GCCCCAGTGGTCTTACATGCACATC-3′; Probe: 5′-(ROX)TGCACCAGACCCGGGCTCAGGTACTCCGA(BHQ2)-3′. All primers and probes were synthesized by Sangon Biotech (Shanghai,China).

### Plaque assay

Cells were seeded in 12-well plates at a density of 4 × 10^5^ cells per well and grown to 90%–95% confluency. After 2 h of infection with 10-fold serially diluted virus, the inoculum was replaced with an agarose overlay mixture (4% agarose and 2 × DMEM medium mixed at 1:3 ratio). The cells were then incubated at 37 °C for 7 days. Cells were fixed with 4% PFA and stained with 1% crystal violet, and plaques were counted to calculate PFU/mL.

### Immunofluorescence assay

Cells were fixed with 4% paraformaldehyde for 30 min and permeabilized with 0.5% Triton X-100 for 1 h at room temperature. Cells were then incubated with the primary antibody against adenovirus (Abcam, Cat. # ab7428) diluted in 1% BSA at 4°C overnight. Following PBST washes, cells were incubated with Alexa Fluor 488-conjugated secondary antibody (Invitrogen, Cat. # A11029) for 1 h at room temperature protected from light. Nuclei were stained with DAPI for 5 min.

### Time-of-compound-addition assay

To evaluate stage-specific antiviral effects, cells were treated with compounds during distinct phases relative to HAdV infection (MOI = 0.05): pre-attachment phase (−2–0 hpi), entry phase (0–2 hpi), immediate early phase (2–48 hpi), early phase (6–48 hpi), late phase (12–48 hpi), and full cycle (−2–48 hpi). All groups were cultured at 37 °C in a 5% CO₂ atmosphere. The replication levels of HAdV were quantified by qPCR at 48 hpi.

### Construction of DHFR-knockdown cell line and rescue by DHFR overexpression

To construct a DHFR-knockdown cell line, a specific sgRNA targeting the DHFR gene (GCGCAGACGCCTGGGAACTG) was designed and cloned into the sgRNA expression plasmid via restriction enzyme digestion and ligation. The A549-CRISPRi cell line, which stably expresses the dCas9-KRAB protein, was used for knockdown experiments. For lentiviral packaging, 293T cells were seeded in 6-well plates at 6 × 10⁵ cells/well and transfected using a plasmid mixture containing the sgRNA expression plasmid and the lentroviral packaging plasmids (provided by Prof. Ruilin Tian’s laboratory). Viral supernatants were collected 48 h post-transfection and used to infect A549-CRISPRi cells. At 24 h post-infection, the culture medium was replaced with complete DMEM medium supplemented with nucleotides to maintain normal cell growth.

To perform rescue experiments, we constructed an sgRNA-resistant DHFR overexpression plasmid (OE-DHFR). For lentiviral packaging, 293T cells were seeded in 6-well plates at 6 × 10⁵ cells/well and co-transfected with the OE-DHFR plasmid together with the packaging plasmids psPAX2 and pMD2.G. Viral supernatants were collected 48 h after transfection. The OE-DHFR lentivirus was then used to transduce sgControl and sgDHFR cell lines. At 48–72 h post-transduction, DHFR expression was assessed by Western blot, and viral replication was evaluated to confirm rescue of the knockdown phenotype.

### Western blotting

Total cellular protein was extracted using RIPA lysis buffer. Equal amounts of protein lysates were separated by 10% SDS-PAGE and transferred to PVDF membranes. After blocking with 5% non-fat milk for 1 h at room temperature, the membranes were incubated with primary antibody against DHFR (OriGene, Cat. # TA500543) at 4°C overnight. Following washes, the membranes were incubated with appropriate secondary antibodies and visualized using the ChemiDoc MP Imaging System (Bio-Rad). Band densities were quantified using ImageJ software.

### Molecular docking

The spatial geometries of compounds AMT, PDX, and MTX were charged and minimized by Acpype server (20). The DHFR model was obtained based on the 4MK6 crystal structure from the PDB website. The protein model was processed using AutoDockTools-1.5.6 to meet general molecular docking requirements, followed by reference ligand docking to validate the model. The energy grid was built in a cubic box measuring 24*24*24 Å. Molecular docking was performed using AutoDock Vina 1.1.2 with 20 docked poses generated for each experiment (21). The results were visualized using Pymol 3.1 software.

### Binding assay

The binding of AMT, MTX, and PDX to human DHFR (hDHFR) was evaluated by surface plasmon resonance (SPR) using a Biacore 8K instrument (Cytiva). Wild-type hDHFR was prepared as described previously (22). hDHFR was immobilized on a CM5 sensor chip by standard amine coupling. AMT, MTX, and PDX were diluted in PBS containing 0.05% (vol/vol) Tween 20 and injected over the chip surface at concentrations ranging from 0.8 to 50 μM at a flow rate of 30 μL/min. The association and dissociation phases were monitored for 90 s and 90 s, respectively. CDV was included as a negative control. The binding kinetic parameters were analyzed using Biacore 8K Evaluation Software, and data plotting was performed with GraphPad Prism 10.0.

### Antiviral drug tests in 3D lung organoids

3D lung organoids were purchased from Shenzhen Synorg Biotechnology Co., Ltd. These alveolar-type lung organoids faithfully recapitulate the key features of human alveolar tissue, shown by the expression of the alveolar epithelial cell markers TTF-1 (Abcam ab125650), SFTPC (Abcam ab90716), and HOPX (Abcam ab230544), as well as the fibroblast marker α-SMA (Abcam ab7817) and the macrophage marker CD68 (Abcam ab955). H&E staining of organoids was performed by Servicebio (Wuhan, China). Organoids were infected with HAdV-B55 at an MOI of 11 for 2 h. Following infection, the inoculum was removed and replaced with medium containing a series of drug concentrations. After 48 hpi, organoids were harvested and digested into single-cell suspensions using 0.25% Trypsin-EDTA. Total RNA was extracted from the cell suspensions using a commercial RNA isolation kit (Qiagen, Cat. # 74106), and viral RNA levels were quantified by qPCR. Dose-response curves based on viral RNA reduction were generated to evaluate antiviral efficacy.

### Measurement of DHFR enzyme activity

After 48 h of treatment with or without compounds, organoids were harvested and dissociated into single-cell suspensions using 0.25% Trypsin-EDTA. Total protein was extracted using a non-denaturing lysis buffer, and DHFR enzyme activity was measured using a commercial kit (Elabscience, Cat. # E-BC-K816-M).

## RESULTS

### Identification of HAdV inhibitors from a library of host-targeting small molecules

**Fig 1:**
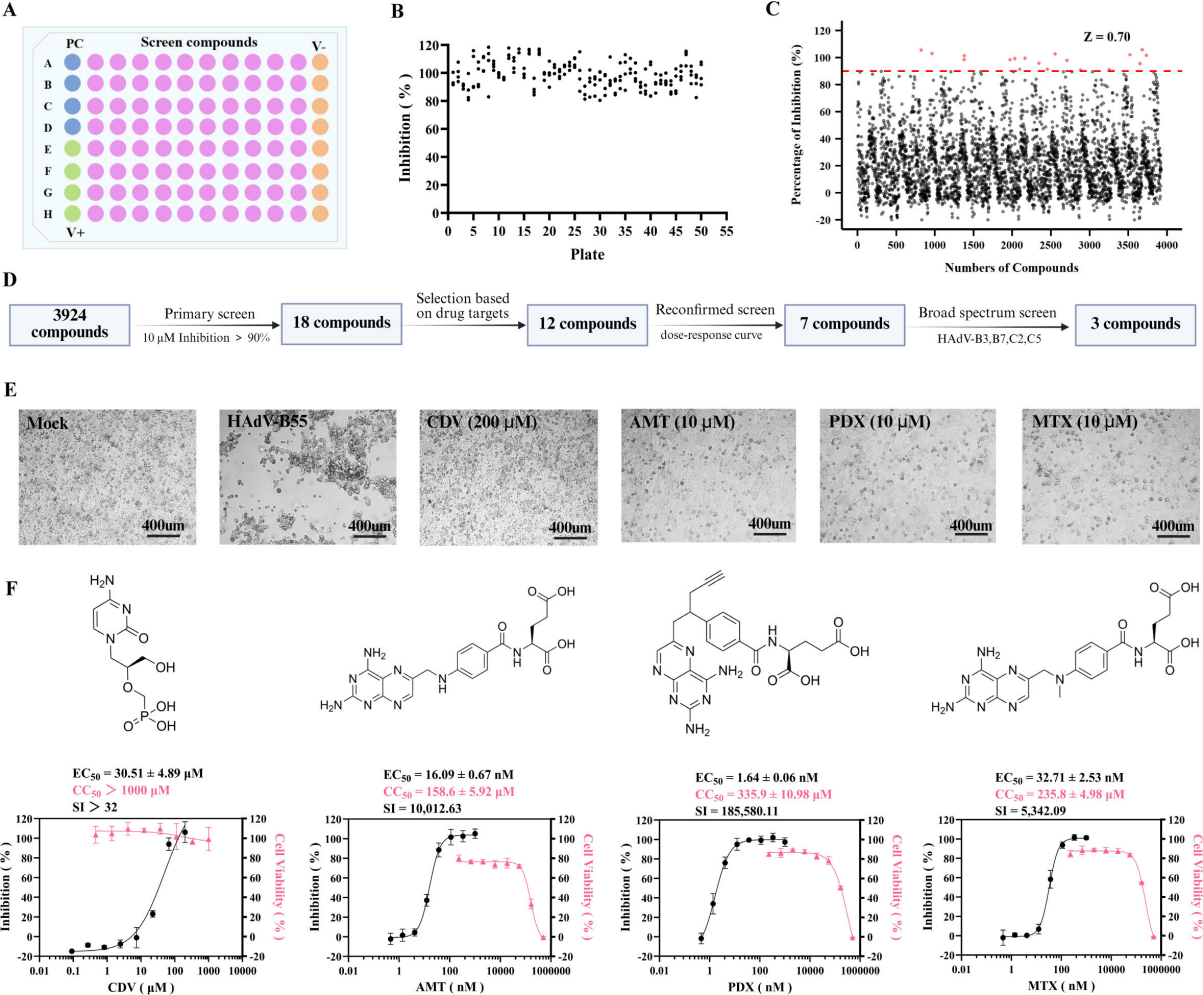
Screening of the compound library for HAdV inhibitors and identification of positive hits. (**A**) Screening layout in 96-well plates. Each plate included a positive control group (PC), a negative control group (V^+^), and a blank control group (V^–^). The positive control group received 200 μM CDV. (**B**) Screening controls. The inhibition rates of the positive control groups across all screening plates are shown. (**C**) Screening results. From a compound library containing 3,924 compounds, 18 compounds (red dots) with inhibition rates greater than 90% were identified. (**D**) Screening workflow. (**E**) CPE in HAdV-B55 infected HEp-2 cells treated with CDV (200 μM), AMT (10 μM), PDX (10 μM), and MTX (10 μM). The scale bar is 400 μm. (**F**) Dose-response curves of compounds against HAdV-B55 infection in HEp-2 cells and the structural formulas of these compounds. Viral inhibition percentage and cell viability were determined by CPE assay. Data are presented as mean ± SD from at least three independent experiments.

We developed a screening assay to identify HAdV inhibitors using HEp-2 cells cultured in 96-well plates and HAdV-B55 as the representative of HAdV (Fig. 1A). The assay utilized CPE as the readout, with 200 μM CDV serving as a positive control, which consistently achieved over 80% inhibition (Fig. 1B). The assay exhibited high robustness with a Z factor of 0.7, confirming its suitability for large-scale screening applications (Fig. 1C). The screening workflow in this study is summarized in Fig. 1D. A library of 3,924 compounds with known or potential activity against host targets was firstly screened at a concentration of 10 μM. Primary screening identified 18 compounds exhibiting greater than 90% inhibition of HAdV-B55, which were designated as primary candidates. Based on drug target relevance and mechanistic profiles, 12 of these candidates were selected for further dose-response evaluation. Dose-response validation experiments identified seven compounds as potential inhibitors of HAdV-B55. Of these, only three folate antagonists, including AMT, PDX, and MTX, exhibited broad-spectrum activity against various common clinical adenovirus serotypes and were, therefore, selected for further study. Compared to CDV (EC_50_ = 30.51 µM), the three folate antagonists exhibited significant antiviral activity against HAdV-B55 at nanomolar concentrations. The compounds AMT, PDX, and MTX significantly inhibited HAdV-B55-induced CPE (Fig. 1E), with EC_50_ values of 16.09 nM, 1.64 nM, and 32.71 nM, respectively (Fig. 1F). Among these, PDX demonstrated the highest SI and the lowest cytotoxicity, indicating superior potential for further therapeutic development.

### Broad-spectrum inhibitory effects of AMT, PDX, and MTX on HAdV replication *in vitro*

**Fig 2:**
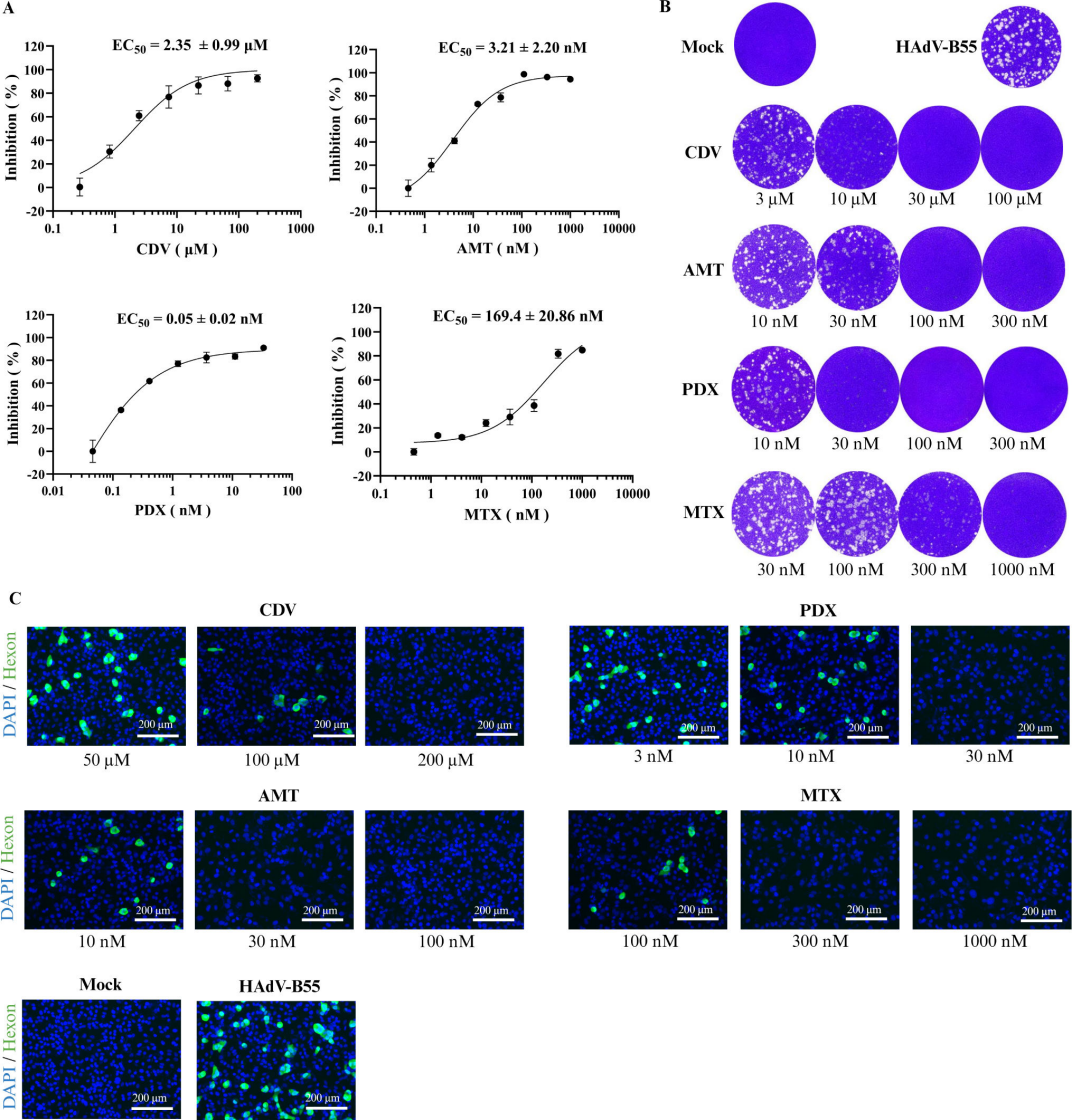
Evaluation and validation of the *in vitro* antiviral activities of AMT, PDX, and MTX against HAdV. (**A**) Dose-response curves of compounds against HAdV-B55 infection in A549 cells. Viral inhibition percentage was quantified by qPCR. Data are presented as mean ± SD from at least three independent experiments. (**B**) Antiviral effects of compounds against HAdV-B55 in plaque assay. Morphological changes were recorded by optical microscopy (scale bar = 400 µm). (**C**) Antiviral effects of compounds against HAdV-B55 in A549 cells. Immunofluorescence images showing viral hexon protein (green) and nuclei (blue) (scale bar = 200 µm).

We further evaluated and confirmed the anti-HAdV activity of three folate antagonists *in vitro* with different evaluation methodology including qPCR, plaque assay and IFA. CDV was consistently used as a positive control throughout the study. In A549 cells, AMT, PDX, and MTX all demonstrated similar anti-viral activity against HAdV-B55 replication when quantified by qPCR assay, with EC_50_ values of 3.21 nM, 0.05 nM, and 169.40 nM, respectively (Fig. 2A). Plaque assays also confirmed that all three antagonists effectively inhibited viral plaque formation in a dose-dependent manner (Fig. 2B). PDX exhibited the most potent activity, completely suppressing plaque formation at 30 nM. In contrast, both AMT and MTX required higher concentrations to achieve complete suppression. Immunofluorescence analysis further confirmed the dose-dependent antiviral effects of these compounds (Fig. 2C). Furthermore, broad-spectrum activity was observed against clinically relevant adenovirus serotypes, including HAdV-B3, B7, C2, and C5 (Table 1 and Fig. S1). Among these antagonists, PDX maintained the highest potency and SI across all tested serotypes, demonstrating superior antiviral activity compared to both AMT and MTX.

**TABLE 1:** AMT, PDX, and MTX show broad-spectrum inhibitory activity of against HAdV B55, B3, B7, C2, and C5 in HEp-2 cells^*a*^

	Compund	EC_50_				
		HAdV-B55	HAdV-B3	HAdV-B7	HAdV-C2	HAdV-C5
1	CDV (μM)	87.56 ± 10.46	13.21 ± 0.79	7.23 ± 2.46	53.21 ± 7.18	14.78 ± 1.53
2	AMT (nM)	4.05 ± 0.12	32.87 ± 3.61	79.74 ± 23.69	35.69 ± 1.29	15.99 ± 4.13
3	PDX (nM)	1.23 ± 0.13	0.69 ± 0.09	1.63 ± 0.53	3.53 ± 0.85	1.42 ± 0.04
4	MTX (nM)	0.31 ± 0.02	38.37 ± 2.15	89.71 ± 13.39	13.19 ± 1.96	14.79 ± 2.29

^*a*^ Viral inhibition percentage was quantified by qPCR. Data are presented as mean ± SD from at least three independent experiments.

### AMT, PDX, and MTX inhibit HAdV-B55 replication by targeting host folate-dependent metabolic pathways

**Fig 3:**
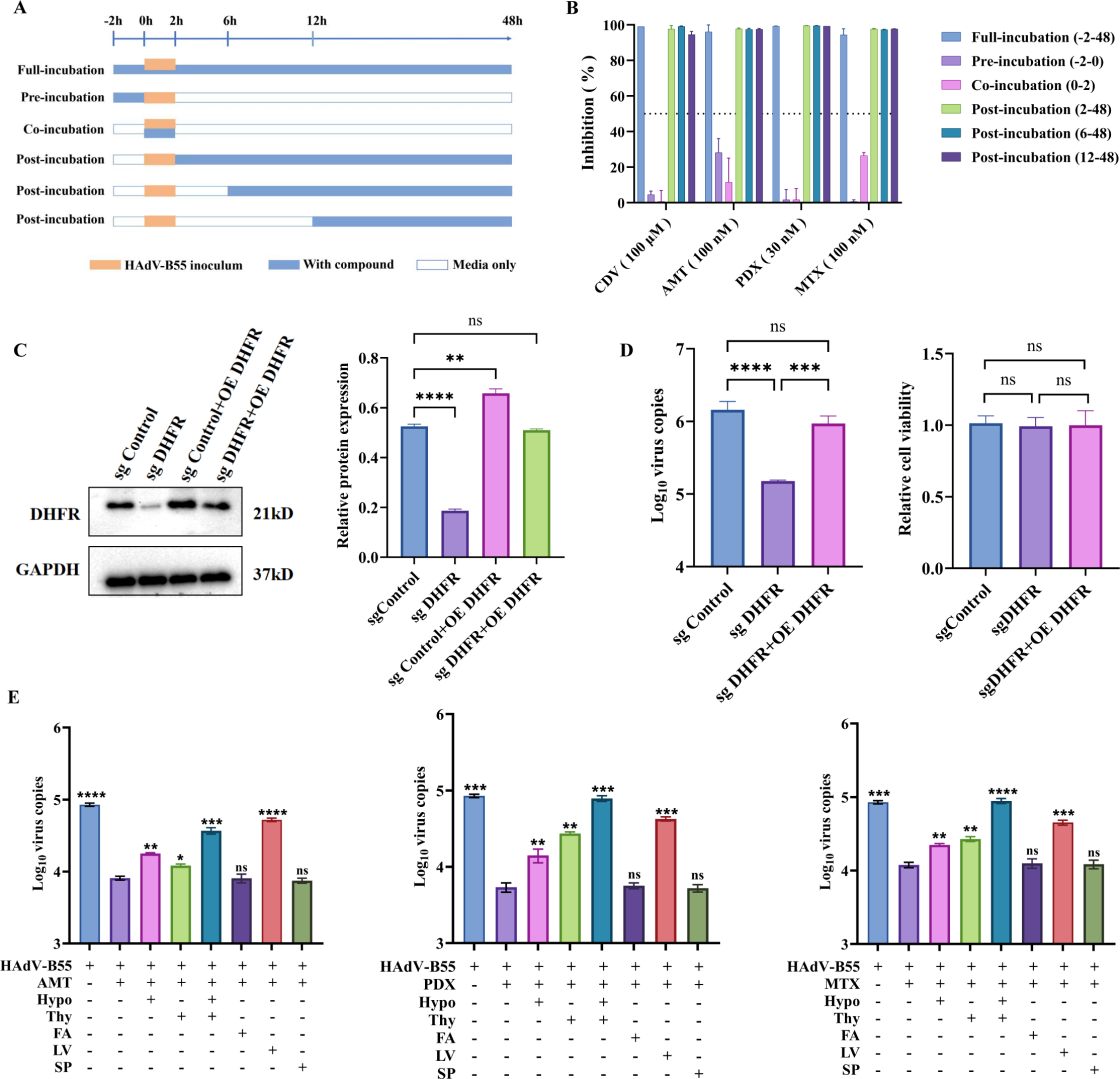
AMT, PDX, and MTX inhibit HAdV-B55 replication through modulation of the folate metabolic pathway. (**A and B**) Time-of-addition assay. HEp-2 cells were infected with HAdV-B55 at an MOI of 0.05, and compounds were added at various time points throughout the infection process. At 48 h post-infection, nucleic acids were extracted from the cell supernatant and viral copy numbers were quantified by qPCR. (**C**) A549 cells were transfected with sgControl or sgDHFR, with or without overexpression of DHFR. Representative immunoblots are shown (left), and band intensities were quantified using ImageJ software (right). Relative protein levels were normalized to GAPDH as a loading control. (**D**) A549 cells were transfected with sgControl or sgDHFR, with or without overexpression of DHFR, and then infected with HAdV-B55. Viral copy numbers were quantified by qPCR (left), and cell viability was assessed by neutral red staining (right). (**E**) A549 cells infected with HAdV-B55 were treated with AMT (100 nM), PDX (30 nM), or MTX (300 nM) and supplemented separately with Hypo (50 μM), Thy (50 μM), FA (50 μM), LV (10 μM), or SP (50 μM). Viral nucleic acids were detected by qPCR, and copy numbers were calculated.Statistical significance was determined by Student’s *t*-test. (*P < 0.05, **P < 0.01, ***P < 0.001, ****P < 0.0001; ns, not significant).

A time-of-addition assay was performed to determine the stage at which folate antagonists exert their antiviral effects against HAdV-B55. Compounds were administered at various time points: 2 h prior to infection, during viral adsorption, and at 2, 6, and 12 hpi, as well as throughout the entire infection cycle (Fig. 3A). The results demonstrated that these compounds significantly inhibited viral replication both during early and late stages of infection (Fig. 3B). Even when administered as late as 12 hpi, folate antagonists still effectively suppressed viral replication. It indicate that folate antagonists may disrupt intracellular metabolic processes during the replication phase of the HAdV infection cycle.

Folate metabolism is central to *de novo* nucleotide biosynthesis, and DHFR serves as a key rate-limiting enzyme in this pathway (23). We, therefore, examined the role of DHFR in viral replication. We knocked down DHFR in A549 cells using CRISPR-dCas9 and rescued its expression with an sgRNA-resistant DHFR construct (Fig. 3C). DHFR knockdown markedly suppressed HAdV replication, while re-expression of DHFR restored viral replication (Fig. 3D), confirming that DHFR is essential for efficient HAdV propagation. Cell viability assays showed that knockdown of DFHR caused no significant cytotoxicity, ruling out non-specific cell death (Fig. 3D).

As competitive inhibitors of DHFR, AMT, MTX, and PDX likely inhibit viral replication by depleting intracellular tetrahydrofolate (THF) pools and thereby blocking the *de novo* nucleotide biosynthesis pathway required for viral replication (24). To elucidate the specific mechanism, we performed metabolic rescue assays using hypoxanthine (Hypo, purine salvage pathway substrate), thymidine (Thy, pyrimidine alvage pathway substrate), folic acid (FA), leucovorin (LV, a downstream metabolite of the DHFR pathway), and sodium pyruvate (SP), during HAdV-B55 infection with the indicated compound (Fig. 3E). The results showed that supplementation with LV, but not FA, effectively rescued the replication of HAdV-B55 when treated with the three compounds (Fig. 3E). Meanwhile, the addition of either Hypo or Thy partially reversed the inhibitory effects of all three compounds on viral replication. Notably, combined supplementation with Hypo and Thy significantly enhanced viral replication under treatment with each of the three antagonists. In contrast, supplementation with SP, a metabolite outside the nucleotide biosynthesis pathway, failed to rescue viral replication. In DHFR-knockdown cells, supplementation with Hypo, Thy, and LV restored viral replication, which is consistent with the rescue observed in compound-treated cells (Fig. S2). These results indicate that the antiviral effect of folate antagonists is associated with the inhibition of DHFR and that the folate-supported *de novo* synthesis pathways for both purines and pyrimidines are critical for viral replication.

### Folate-like binding of AMT, PDX, and MTX within the DHFR catalytic pocket

**Fig 4:**
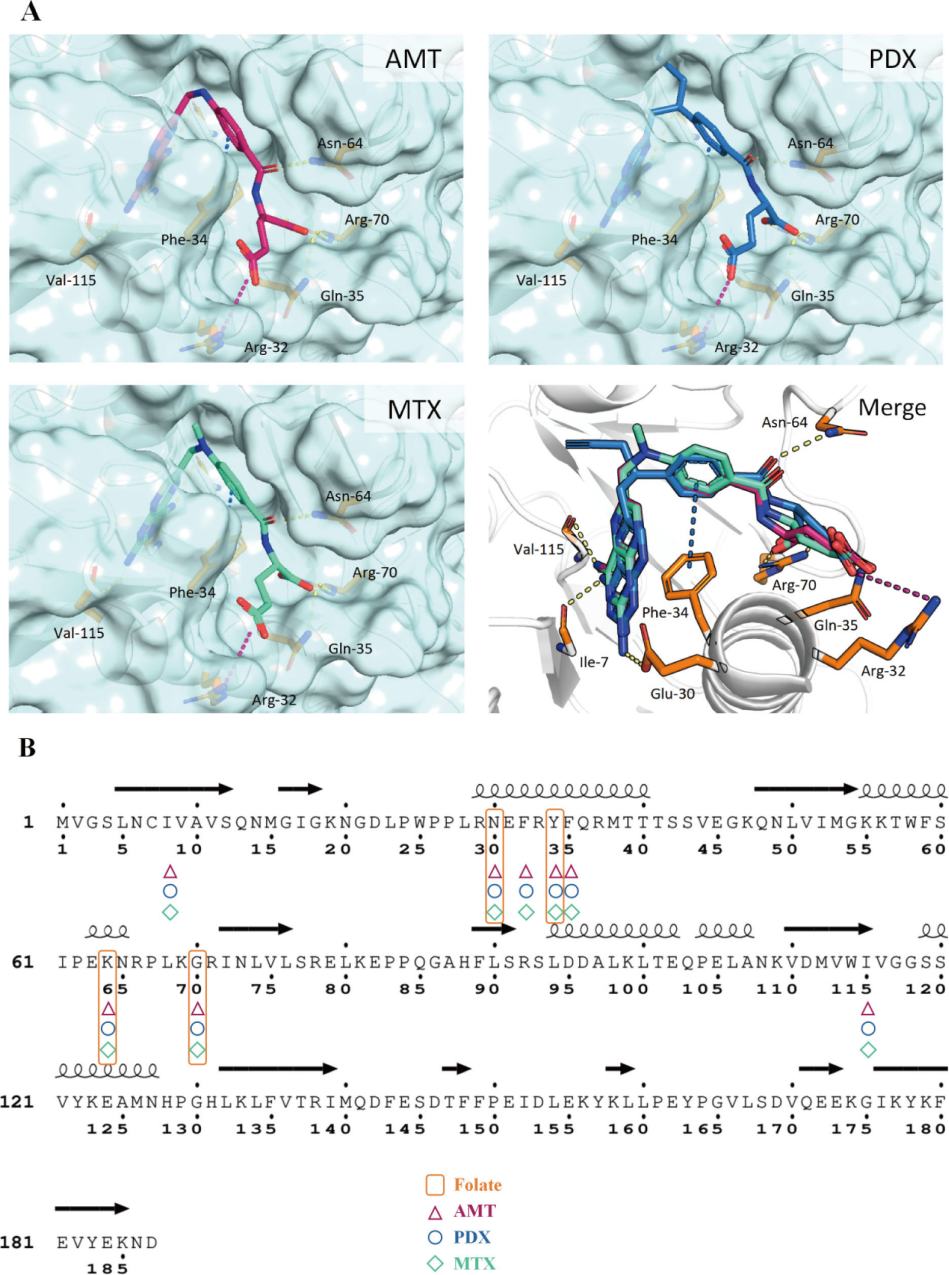
Binding of AMT, PDX, and MTX to the DHFR catalytic pocket mimics that of folate. (**A**) Molecular docking results depicting the binding conformations of AMT, PDX, and MTX within the DHFR active site. The individual ligand-receptor complexes and their structural superposition reveal a shared binding pose, with all three compounds occupying the catalytic pocket and forming specific interactions with key amino acid residues. (Orange: key amino acid residues; Yellow dash line: H-bond; Blue dash line: π-π stacking interactions; Magenta dash line: salt-bridge interactions.) (**B**) Sequence alignment of AMT, PDX, MTX, and the natural substrate folate. The DHFR model was based on the PDB: 4M6K. Docking simulations were performed using AutoDock Vina 1.1.2, and the model was validated by re-docking the reference ligand. All structural visualizations were prepared using PyMOL 3.1.

To investigate the mechanism of the antiviral activity of AMT, PDX, and MTX, we performed molecular docking analyses using the DHFR crystal structure (PDB: 4M6K). Docking simulations were conducted with AutoDock Vina 1.1.2, and the docking protocol was validated by re-docking the reference ligand (Fig. 4A). The results showed that AMT, PDX, and MTX share a highly similar binding mode with folate, all stably positioned within the active pocket of DHFR and forming comparable interactions with key residues N30, Y34, K64, and G70 (Fig. 4B). We further evaluated binding affinities to human DHFR by SPR. AMT, PDX, and MTX all bound to hDHFR with dissociation constants (Kd) of 5.77 nM, 23.8 nM, and 2.9 nM, respectively. No binding signal was detected for the negative control CDV (Fig. S3). These results suggest that AMT, PDX, and MTX inhibit DHFR through competitive binding. Given the essential role of DHFR in purine and thymidine biosynthesis, its inhibition is expected to reduce the availability of nucleotides in host cells, thereby indirectly limiting the nucleic acid synthesis required for viral replication.

### PDX inhibited HAdV replication in 3D lung organoid model

**Fig 5:**
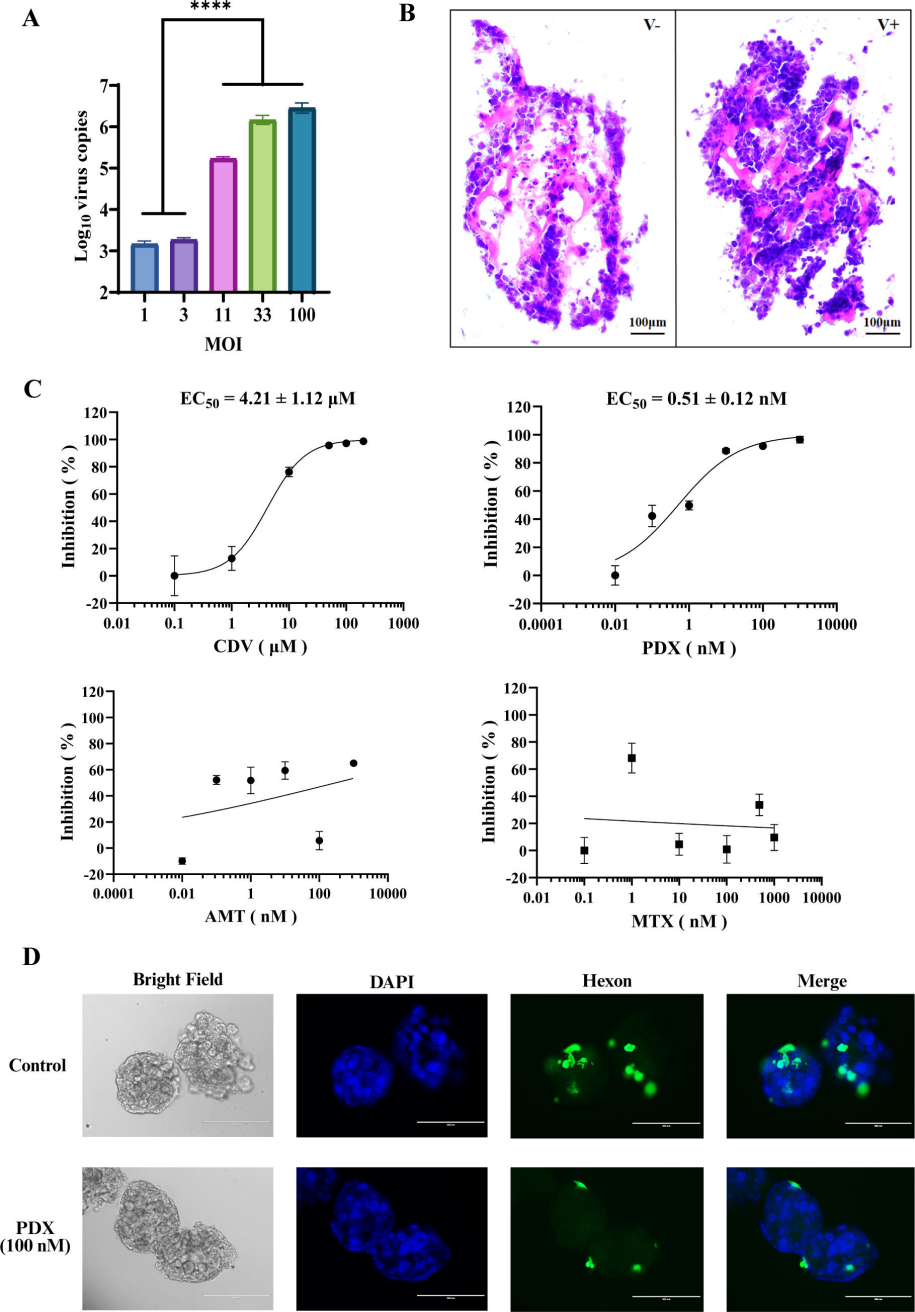
PDX show antiviral activity against HAdV in a 3D lung organoid model. (**A**) Replication of HAdV-B55 in 3D lung organoids at different MOI. Viral RNA levels were quantified by qPCR, and copy numbers were calculated. (**B**) H&E staining of lung organoids to evaluate HAdV-B55-induced pathological changes. Mock-infected (left) and virus-infected (right, MOI = 11) organoids are shown (scale bar = 100 µm). (**C**) Dose-response curves of the compounds in a 3D lung organoid model. Organoids were infected with HAdV-B55 at an MOI of 11 and treated with serially diluted compounds. Viral RNA levels were quantified by qPCR after 48 h. Data are presented as mean ± SD from at least twice independent experiments. (**D**) Antiviral effect of 100 nM PDX visualized by immunofluorescence. Representative images show viral hexon protein (green) and nuclei (blue) (scale bar = 400 µm). Statistical significance was determined by Student’s *t*-test (****P < 0.0001).

To determine whether HAdV-B55 could efficiently infect lung organoids, we first assessed viral replication at different MOIs and found that viral replication increased in a dose-dependent manner as MOI rose (Fig. 5A). H&E staining of organoid sections revealed that mock-infected organoids exhibited large, multilocular complex structures with abundant and well-defined luminal spaces. Cells were arranged along the luminal boundaries with regular morphology, collectively preserving an alveolar-like three-dimensional architecture. In contrast, HAdV-B55-infected organoids displayed markedly denser and more solid cellular aggregates, with a significant reduction in both the number and size of luminal spaces. Focal regions showed irregular cell morphology and evidence of cellular fragmentation (Fig. 5B). These findings confirm that the model recapitulates virus-induced tissue damage. We then evaluated the antiviral activity of three folate antagonists against HAdV-B55 in the lung organoid model. CDV was used as a positive control and exhibited potent inhibition (EC_50_ = 4.21 µM) in organoids. Among the three folate antagonists, only PDX demonstrated significant antiviral activity in organoids with an EC_50_ of 0.51 nM, while AMT and MTX showed no antiviral activity in the same organoid model (Fig. 5C). Furthermore, immunofluorescence assay using an anti-HAdV hexon antibody confirmed that 100 nM PDX effectively suppressed HAdV-B55 replication in organoids (Fig. 5D). In pan-serotype assays using lung organoids, PDX also showed potent antiviral activity against HAdV-B3, B7, C2, and C5 (Fig. S5).

To confirm the expression and functional role of DHFR in the lung organoid model, we assessed DHFR protein levels by Western blot. DHFR was detectable in untreated lung organoids. PDX treatment significantly upregulated DHFR protein expression (Fig. S6A), likely as a compensatory response to DHFR inhibition (25). Similarly, PDX treatment markedly reduced DHFR enzymatic activity (Fig. S6B), confirming that it effectively inhibits DHFR catalytic function in this model. These results suggest that the antiviral activity of PDX depends on inhibition of DHFR.

## DISCUSSION

Folate metabolism is a critical biochemical process for cell proliferation and genetic material synthesis, providing essential one-carbon units for the *de novo* synthesis of purines and thymidylate (24). Folate antagonists are a class of drugs that competitively inhibit key enzymes in folate metabolism, thereby blocking nucleotide synthesis (26). In this study, we identified three folate antagonists from an approved drug library with antiviral activity against HAdV: AMT, PDX, and MTX. As a first-generation folate antagonist, AMT is a potent DHFR inhibitor and was historically used in the treatment of acute leukemia. However, due to its narrow therapeutic window and high toxicity, AMT has largely been replaced by MTX (27). MTX, a 4-amino-10-methyl derivative of AMT, offers a superior therapeutic index and better tolerability, making it a cornerstone therapy for various cancers, autoimmune diseases, and dermatological conditions (28, 29). PDX is a structurally optimized next-generation folate antagonist approved in 2009 for the treatment of relapsed or refractory peripheral T-cell lymphoma (30). Compared to MTX, PDX exhibits higher affinity for the reduced folate carrier (RFC), enabling more efficient cellular uptake. Following intracellular polyglutamylation, it persistently inhibits DHFR activity, leading to tetrahydrofolate depletion and disruption of nucleotide synthesis (31). Our results demonstrated that PDX significantly inhibits HAdV replication in a dose-dependent manner and exhibits the highest SI among the three compounds. Compared to AMT and MTX, PDX showed markedly improved efficacy (EC_50_ = 1.64 nM) against HAdV along with relatively lower cytotoxicity (CC_50_ = 335.9 µM).

Time-of-addition experiments revealed that all three folate antagonists inhibited viral infection at both early and late stages of the cycle, indicating their interference with multiple phases of viral replication. Subsequent rescue experiments showed that supplementation with FA alone was insufficient to reverse the antiviral effects of these antagonists, whereas the addition of LV effectively restored viral replication. The results are consistent with that the antagonists competitively inhibit DHFR, the enzyme responsible for converting folate to its active form (32–34). Consequently, supplementing the substrate (FA) cannot circumvent the enzymatic blockade (35, 36). As a critical rate-limiting enzyme in the folate metabolic pathway, DHFR is indispensable for intracellular one-carbon metabolism and *de novo* nucleotide synthesis (23). In the present study, concurrent supplementation with Hypo and Thy significantly alleviated the inhibitory effects of the folate antagonists on HAdV-B55 replication. Additionally, HAdV-B55 replication was markedly impaired in cells with low DHFR expression. Collectively, these findings suggest that folate antagonists exert their antiviral activity by suppressing DHFR function, which depletes intracellular purine and pyrimidine nucleotide pools and thereby disrupts the supply of essential precursors required for viral genome replication.

Furthermore, we evaluated the anti-HAdV-B55 activity of these three folate antagonists in a 3D lung organoid model. Although all three compounds showed antiviral effects in cell-based models, only PDX maintained significant inhibitory activity (EC_50_ = 0.51 nM) in the 3D organoid system. This discrepancy may be attributed to the greater physiological relevance of organoids, which more closely mimic the architectural complexity and cellular heterogeneity of *in vivo* tissues. Although AMT, PDX, and MTX share a similar mechanism of function, PDX has been rationally designed to achieve fundamental improvements in drug transport and intracellular retention (31, 37). PDX was engineered to exhibit high affinity for RFC-1, the primary transporter for folate and antifolate uptake into cells (37, 38). Data indicate that PDX’s affinity for RFC-1 (*K*ₘ =0.3 µM) is more than 10-fold greater than that of MTX (*K*ₘ =4.8 µM), enabling PDX to penetrate the 3D structure of organoids more effectively and achieve therapeutic intracellular concentrations (38). Once inside the cell, antifolates must be polyglutamated by folylpolyglutamate synthase (FPGS) to be efficiently retained and exert long-term effects (39, 40). PDX serves as a superior substrate for FPGS, with a polyglutamation efficiency (*V*ₘₐₓ/*K*ₘ = 23.2) significantly higher than that of MTX (*V*ₘₐₓ/*K*ₘ = 2.2), which markedly prolongs its intracellular activity (37, 38). Consequently, the far superior efficacy of PDX over AMT and MTX in organoid models may attribute to the unique pharmacokinetic properties. Furthermore, in this organoid model, PDX treatment led to compensatory DHFR upregulation and reduced enzymatic activity, further confirming that its antiviral activity is mediated through DHFR inhibition (25).

PDX is an FDA-approved drug for the treatment of peripheral T-cell lymphoma, and its well-established clinical profile may facilitate repurposing efforts (30). However, its clinical use is associated with certain adverse effects, including dose-limiting toxicities such as mucositis, reversible thrombocytopenia, and anemia (41, 42). These side effects are more frequently observed during long-term oncology treatments but are likely to be less concerning in the context of acute viral infection due to the significantly shorter treatment duration. For clinical translation, the dosing strategy for PDX needs optimization for acute viral infections. The intermittent high-dose regimen used for lymphoma may not be suitable for short-term antiviral therapy (43). Instead, a new regimen consisting of a short loading dose followed by maintenance dosing should be explored, guided by viral replication kinetics. This approach would rapidly achieve therapeutic concentrations while minimizing toxicity accumulation (38). Furthermore, attention should be paid to the pharmacokinetics and potential drug-drug interactions when PDX is co-administered with commonly used anti-infective agents and immunomodulators, particularly in immunocompromised populations requiring polypharmacy (44). Moreover, existing safety data are primarily derived from cancer patients, whereas populations at high risk for HAdV infection, such as children and transplant recipients, have distinct metabolic profiles and comorbidity backgrounds (38). Therefore, individualized treatment monitoring strategies should be established for these special populations.

Compared with the existing anti-HAdV drugs CDV and BCV, PDX offers several clear advantages: (i) in terms of antiviral potency, the EC_50_ of PDX against HAdV is substantially lower than that of CDV, exhibiting significant antiviral activity at nanomolar concentrations; (ii) PDX follows a different metabolic pathway from CDV and does not depend on active renal tubular secretion, markedly lowering the risk of nephrotoxicity (45, 46); (iii) CDV has poor oral absorption and requires intravenous administration. Although PDX is currently available mainly as an injectable, its optimized structure allows higher cellular uptake and longer intracellular retention, providing a basis for future oral formulations (37, 38); (iv) PDX can cause gastrointestinal toxicities such as mucositis in clinical use, but supplementation with leucovorin and vitamin B12 has been shown to reduce these toxicities without compromising efficacy, offering a practical strategy for managing side effects (47–49).

Host folate and one-carbon metabolism is a promising target for antiviral therapy (24, 50). This pathway includes multiple enzymatic steps beyond DHFR, such as SHMT1/2, TYMS, and enzymes involved in purine biosynthesis. Targeting different steps with combination strategies may therefore improve antiviral efficacy (23, 51). Zhang et al. recently showed that both MTX and the SHMT1/2 inhibitor SHIN1 suppress SARS-CoV-2 replication (24). This supports dual targeting of the folate pathway. Similarly, combining PDX with other folate pathway inhibitors could have synergistic effects against HAdV. Such combinations may improve antiviral potency while reducing individual drug doses and toxicity. Future studies on these combination strategies could help develop host-directed therapies for HAdV infections.

Nonetheless, our study has limitations: (i) the drug efficacy and mechanism of action were primarily established *in vitro*, and future validation in animal models of HAdV infection is necessary; (ii) head-to-head comparisons with clinically relevant anti-HAdV agents, such as BCV, were not performed, leaving the relative potency and potential advantages of PDX to be determined; (iii) although the rescue experiments confirmed the involvement of the folate pathway, further investigation is required to explore other potential non-folate-dependent mechanisms.

In summary, the folate antagonist PDX can be repurposed for the treatment of HAdV infection. As a host-targeted antiviral strategy, PDX offers a high barrier to resistance and broad-spectrum potential. Supported by its established clinical profile and manageable toxicity, PDX represents a promising candidate for the treatment of HAdV infections.

## SUPPLEMENTAL MATERIAL

Supplemental material aac.01960-25-s0001.docx Fig. S1 to S6.

ASM does not own the copyrights to Supplemental Material that may be linked to, or accessed through, an article. The authors have granted ASM a non-exclusive, world-wide license to publish the Supplemental Material files. Please contact the corresponding author directly for reuse.
