# Supplementary material for: Pralatrexate is a potent pan-serotype human adenovirus inhibitor through suppression of dihydrofolate reductase
Source: Antimicrob Agents Chemother. 2026 May 28;70(7):e01960-25. doi: 10.1128/aac.01960-25 (PMC13321789; doi:10.1128/aac.01960-25)
Supplement: Supplemental material — Fig. S1 to S6. [file aac.01960-25-s0001.docx]

**Supplementary Fig. 1. Pan-serotype inhibitory effects of AMT, PDX, and MTX on HAdV replication in A549 cells.**

**

**

Dose-response curves of the compounds against HAdV-B55, B3, B7, C2, and C5. Viral inhibition percentage was quantified by qPCR. Data are presented as mean ± SD from at least three independent experiments.

**Supplementary Fig. 2. Effects of folate metabolism compounds on HAdV replication in DHFR-knockdown cells.**


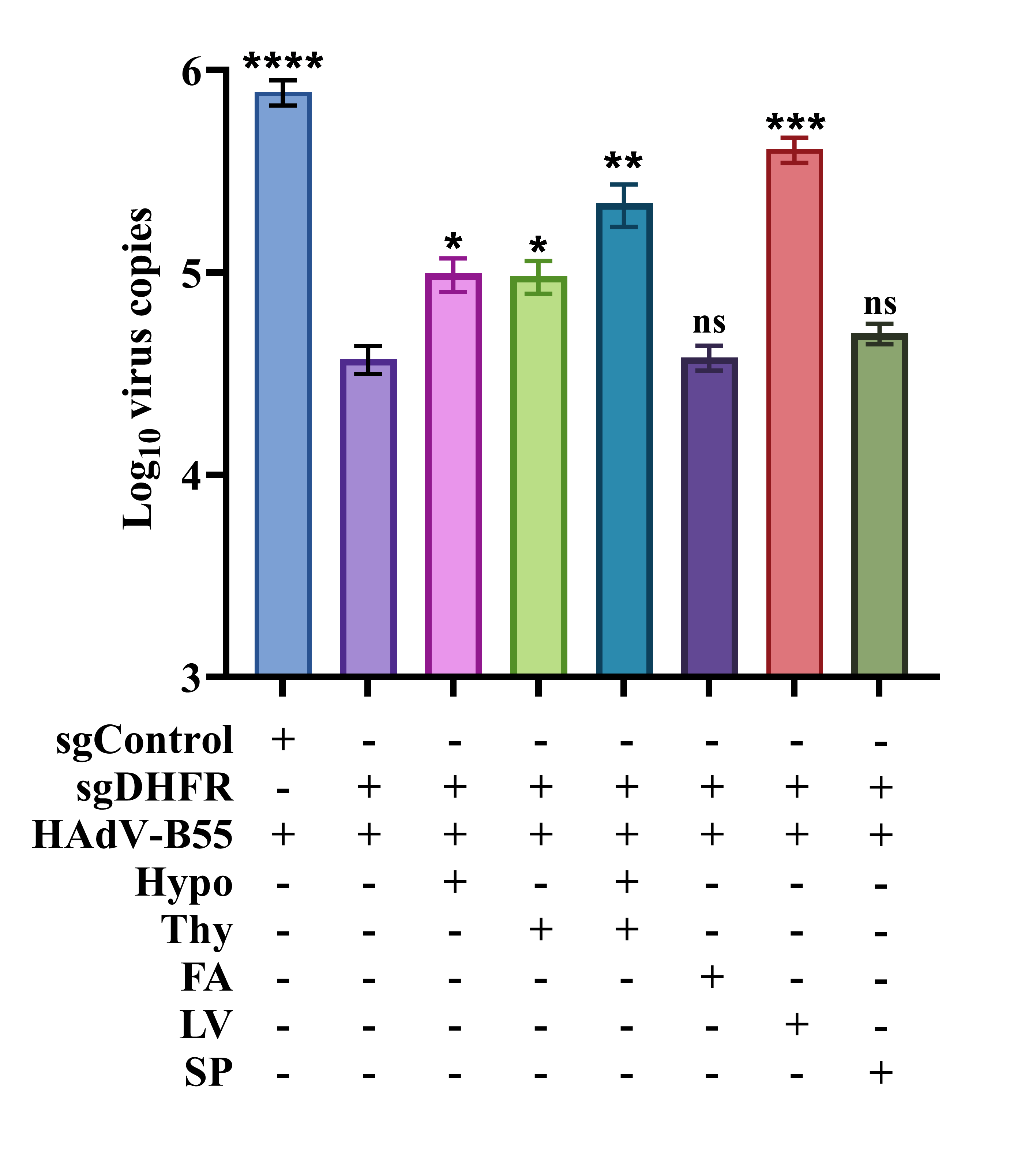


A549 cells transduced with sgControl or sgDHFR were infected with HAdV-B55 and supplemented separately with Hypo (50 μM), Thy (50 μM), FA (50 μM), LV (10 μM) or SP (50 μM). Viral nucleic acids were detected by qPCR and copy numbers were calculated. Statistical significance was determined by Student’s t-test (**P* < 0.05, ***P* < 0.01, ****P* < 0.001, *****P* < 0.0001; ns, not significant).

**Supplementary Fig. 3. Binding affinities of AMT, PDX, and MTX to hDHFR measured by SPR.**


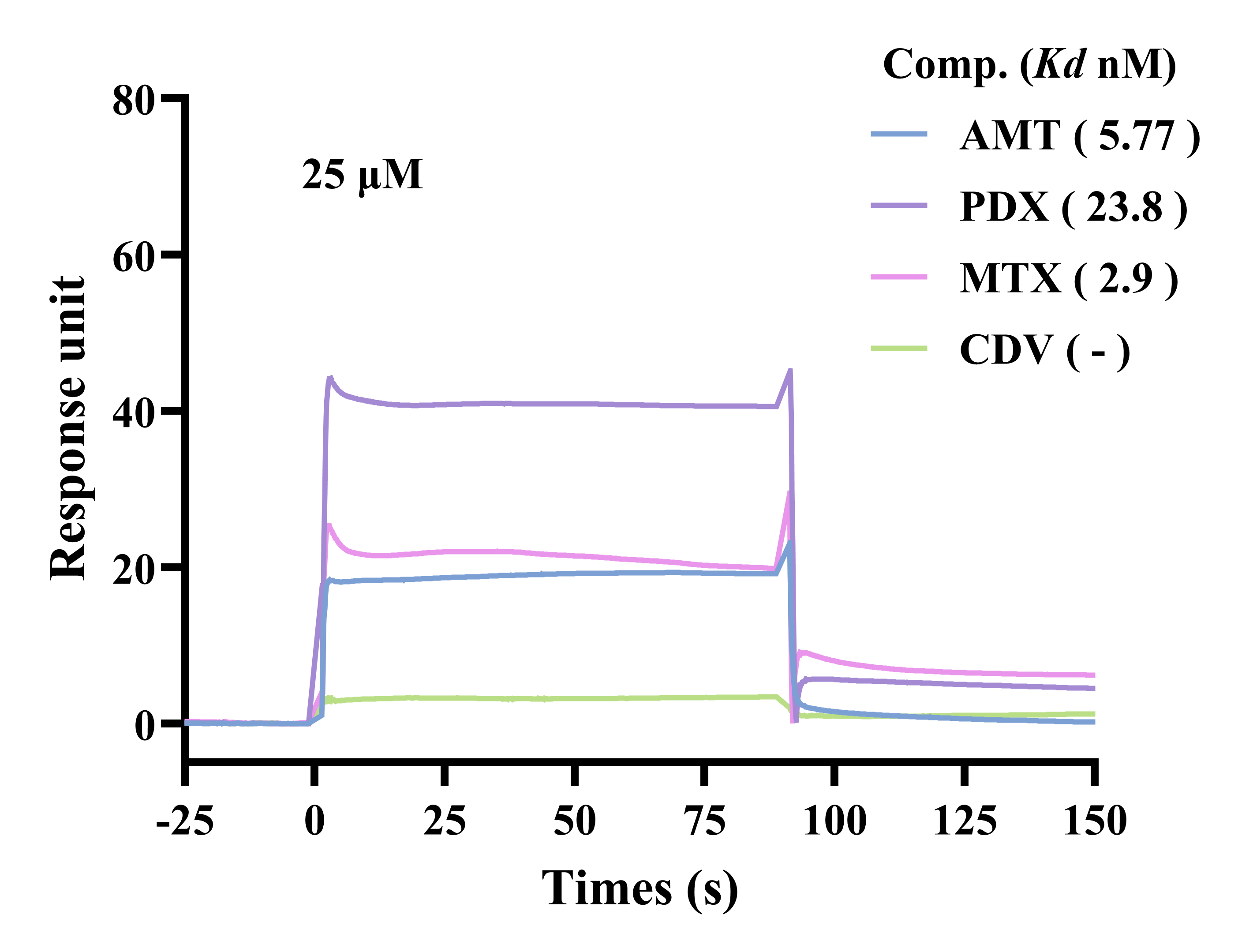


SPR analysis of AMT, MTX, and PDX binding to immobilized hDHFR. Representative sensorgrams and calculated Kd values are shown. CDV was used as a negative control.

**Supplementary Fig. 4. Morphological and phenotypic characterization of human alveolar organoids.**


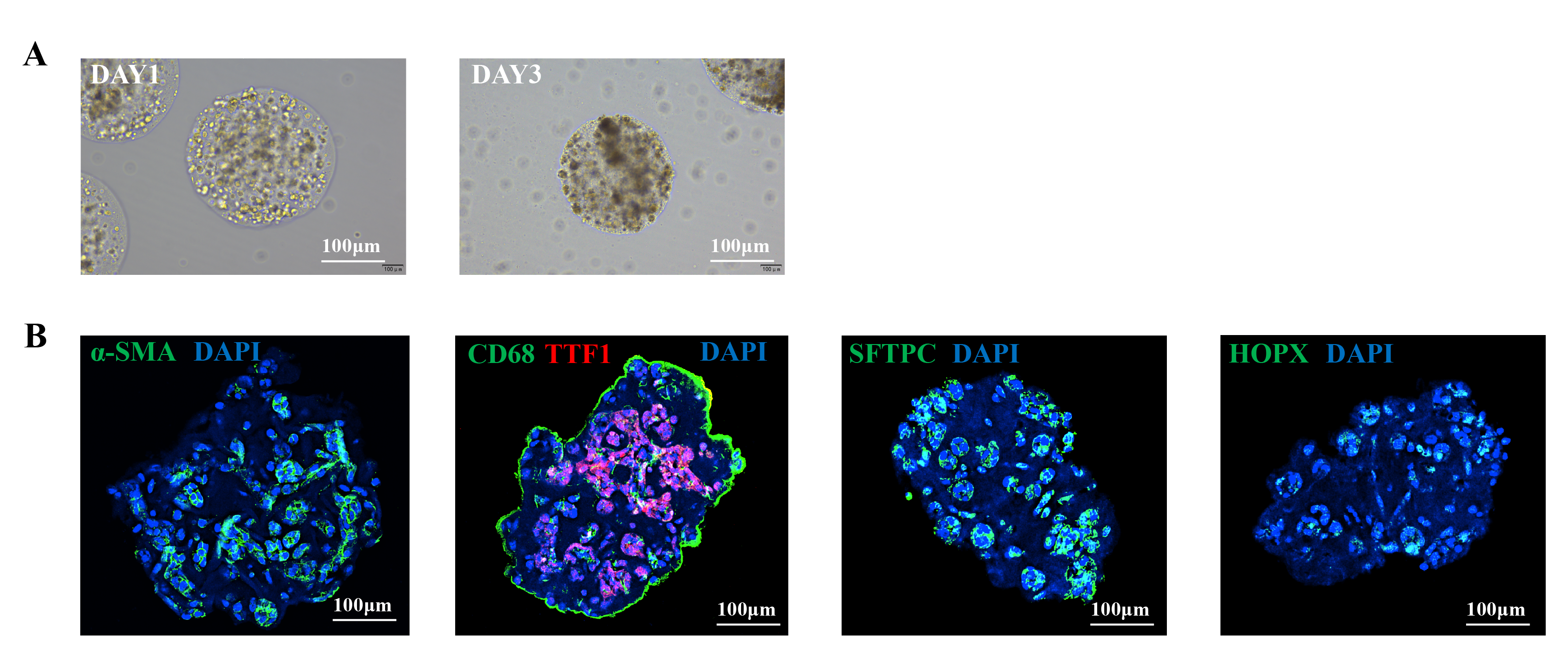


(A) Bright-field images showing organoid morphology on days 1 and 3 of culture (scale bar = 100 μm). (B) Immunofluorescence images showing expression of α-SMA (green), CD68 (green), TTF1 (red), SFTPC (green), HOPX (green), and DAPI (blue) in organoids (scale bar = 100 μm).

**Supplementary Fig. 5. Pan-serotype inhibitory effects of AMT, PDX, and MTX on HAdV replication in lung organoids.**

**
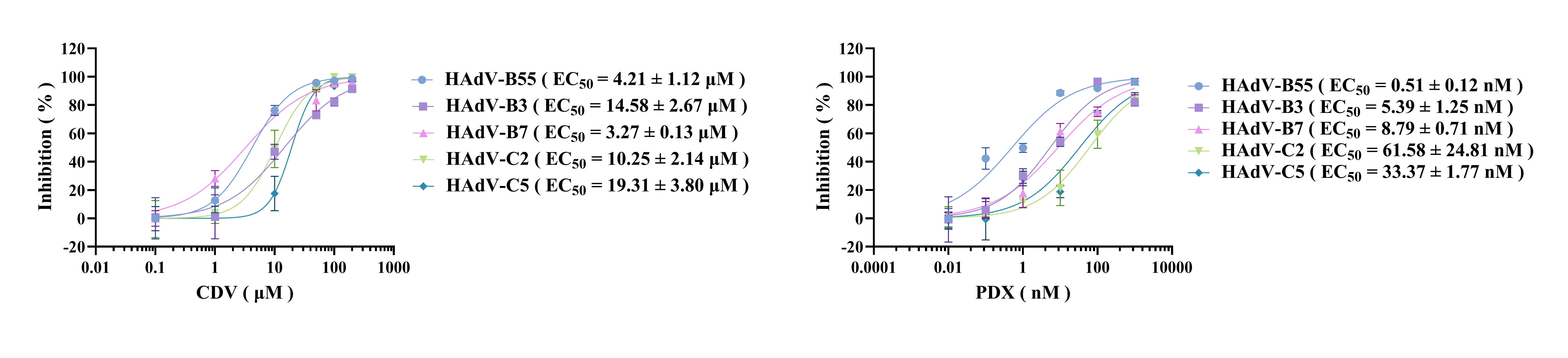
**

Dose–response curves of the compounds against HAdV-B55, B3, B7, C2, and C5. Viral inhibition was quantified by qPCR. Data represent the mean ± SD from at least three independent experiments.

**Supplementary Fig. 6. Validation of DHFR involvement in lung organoids.**

**
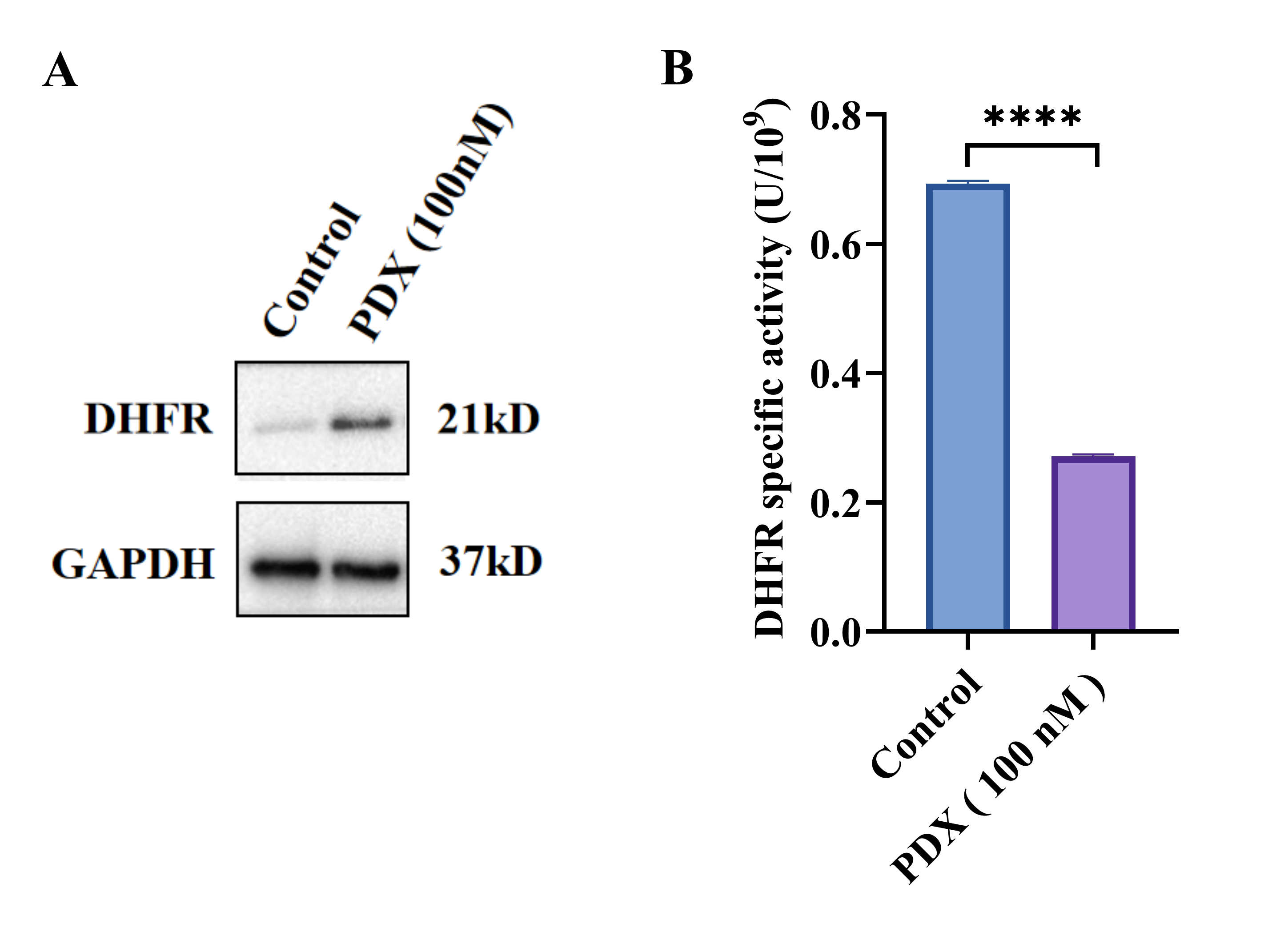
**

(A) Western blot analysis of DHFR expression in lung organoids treated with or without PDX (100 nM). (B) DHFR enzymatic activity in lung organoids treated with or without PDX (100 nM). Statistical significance was determined by Student’s t-test ( *****P* < 0.0001).
